# Sirolimus is efficacious in treatment for extensive and/or complex slow-flow vascular malformations: a monocentric prospective phase II study

**DOI:** 10.1186/s13023-018-0934-z

**Published:** 2018-10-29

**Authors:** Jennifer Hammer, Emmanuel Seront, Steven Duez, Sophie Dupont, An Van Damme, Sandra Schmitz, Claire Hoyoux, Caroline Chopinet, Philippe Clapuyt, Frank Hammer, Miikka Vikkula, Laurence M. Boon

**Affiliations:** 1 Center for Vascular Anomalies, Division of Plastic Surgery, Cliniques universitaires Saint Luc, University of Louvain, 10 avenue Hippocrate, B-1200 Brussels, Belgium; 2 Center for Vascular Anomalies, Institut Roi Albert II, Department of Medical Oncology, Cliniques universitaires Saint Luc, University of Louvain, Brussels, Belgium; 3 Department of Pediatric Hemato-oncology, Cliniques universitaires Saint Luc, University of Louvain, Brussels, Belgium; 4 Center for Vascular Anomalies, Department of Pediatric Hemato-oncology, Cliniques universitaires Saint Luc, University of Louvain, Brussels, Belgium; 5 Center for Vascular Anomalies, Department of Head and Neck Surgery, Cliniques universitaires Saint-Luc, University of Louvain, Brussels, Belgium; 6 Department of Pediatric Hemato-oncology, CHR Citadelle, Liège, Belgium; 7 Department of Pediatric Cardiology, CHRU Lille, Lille, France; 8 Division of Pediatric Radiology, Cliniques universitaires Saint-Luc, University of Louvain, Brussels, Belgium; 9 Division of Interventional Radiology, Cliniques universitaires Saint-Luc, University of Louvain, Brussels, Belgium; 10 Human Molecular Genetics, de Duve Institute, University of Louvain, Brussels, Belgium

**Keywords:** Sirolimus, Rapamycin, Venous malformation, Lymphatic malformation, Complex vascular malformation, Slow-flow anomaly, Extensive vascular anomaly

## Abstract

**Background:**

Extensive and complex vascular malformations often cause chronic pain and severe functional restraint. Conventional treatments, such as surgery and/or sclerotherapy, are rarely curative, underscoring the great need for new therapeutic modalities. Recent preclinical and clinical data demonstrated that sirolimus could offset the progression of vascular malformations and significantly improve quality of life of patients through inhibition of the Phosphatidylinositol-3-kinase (PI3K)/AKT/mammalian Target of Rapamycin (mTOR) pathway. The purpose of this prospective study was to assess the efficacy and safety of this treatment in patients with extensive or complex slow-flow vascular malformations.

**Methods:**

Sirolimus was administered orally on a continuous dosing schedule with pharmacokinetic-guided target serum concentration level of 10 to 15 ng/ml. Patients were seen every month for the first three months and subsequently every three months. The primary endpoints were safety and efficacy, based on clinical, biological and radiological evaluations, as well as a quality of life questionnaire.

**Results:**

Nineteen patients, from 3 to 64 years old, with lymphatic (LM), venous (VM) or complex slow-flow malformations, refractory to standard care, were enrolled and received sirolimus continuously. After 12 months of follow-up, 16 patients were available for assessment of efficacy and safety: all had a significant and rapid improvement of their symptoms and quality of life. In two patients, sirolimus treatment permitted sclerotherapy and surgery, initially evaluated unfeasible. Sirolimus was well tolerated, with mucositis as the most common (10% of patients) grade 3 adverse event.

**Conclusions:**

Sirolimus was efficient in extensive LM, VM and/or complex malformations that were refractory to conventional treatments and was well tolerated.

## Background

Vascular malformations are rare structural anomalies of blood and lymphatic vessels. Depending on the affected vessel type, these malformations are divided into capillary, venous, lymphatic, arteriovenous or combined malformations [1–3]. They are usually present at birth but can appear during childhood or adulthood [4].

Venous malformations (VM) often cause deformation, pain, chronic anaemia and important functional restraint. About 50% of VMs have associated coagulation abnormalities with high D-dimer and normal-to-low fibrinogen levels. This localized intravascular coagulopathy is relatively specific for VM and is used as a diagnostic test [5–8].

Lymphatic malformations (LM) are micro- or macrocystic, or mixed, and can be localized or diffuse, as observed in generalized lymphatic anomalies (GLA). They can cause deformation, infiltration and compression of vital structures, similar to VM. They are often associated with oozing, bruizing and chronic infections [9].

Combined malformations also exist. Typical are the capillary-lymphatico-venous malformations with or without overgrowth of the affected limb (formerly known as Klippel-Trenaunay syndrome, KTS) and the complex vascular malformations (e.g. arterio-venous or venous malformations) seen in the PTEN hamartoma tumour syndrome (PHTS) [10, 11].

The diagnosis of a vascular malformation is usually based on history and physical examination. Further investigations include Doppler ultrasonography, MRI and D-dimer and fibrinogen level measurements to identify a venous component and/or to exclude a coagulation abnormality [12]. Sclerotherapy alone or in combination with surgical resection is the gold standard procedure for most slow-flow vascular malformations [13, 14]. These procedures are rarely curative in patients with extensive and infiltrating lesions. Thus, an important unmet medical need exists.

The tyrosine kinase receptor TIE2 and its ligands angiopoietin-1 and -2, play a key role in vascular maturation and stability through the Phosphatidylinositol-3-kinase (PI3K)/AKT/mammalian Target of Rapamycin (mTOR) signalling pathway. Up to 60% of VMs are due to somatic *TIE2* mutations, resulting in ligand-independent activation of the receptor [15–20]. Another 20% of VMs are due to somatic activating mutations of *PIK3CA* encoding for the p110 catalytic subunit of PI3K [21]. These mutations lead to a sustained activation of the PI3K/AKT/mTOR pathway, responsible for accumulation of endothelial cells due to reduced apoptosis and defective recruitment of vascular smooth muscle cells [15, 22–24], which results in the enlarged, convoluted venous channels. Recently, the first animal model of VM was developed through injection of TIE2-mutated human umbilical vein endothelial cells HUVECs subcutaneously into nude mice. In this model, sirolimus administration stopped the growth of VM-lesions [22]. Similarly, somatic/mosaic *PIK3CA* mutations are found in the majority of LM and complex malformations (KTS), underscoring mTOR activation as an interesting target for medical treatment of slow-flow vascular malformations [22].

Sirolimus, also known as rapamycin, is an allosteric inhibitor of mTOR. It is used in a number of medical disciplines, especially as an immunosuppressive drug to prevent organ rejection, as an anti-angiogenic medication on coated cardiologic stents and as a cytostatic agent in breast and renal cancer. Moreover, our pilot study and two previously reported trials showed efficacy in selected vascular malformations [22, 25, 26]. We now performed a monocentric prospective phase II clinical trial to evaluate sirolimus efficacy and safety for patients (children and adults) with extensive and/or complex slow-flow vascular malformations that were refractory to standard treatments.

## Methods

### Patients

Inclusion criteria included symptomatic slow-flow vascular malformations that were refractory to standard care, such as sclerotherapy and/or surgical resection. Chronic pain, functional restraint, recurrent infections (defined as > 3 episodes/year), oozing, bleeding and/or coagulation abnormality were the symptoms considered for inclusion in the study.There was no age limitation. The eligible patients had to have adequate liver (bilirubin, ASAT, ALAT), medullar (neutrophils ≥ 1500/mm^3^, hemoglobin ≥ 8,0 mg/dl and platelets ≥ 50.000/mm^3^) and renal function (clearance ≥ 70 ml/min/1.73m^2^) with a Karnofsky performance status ≥ 50. If partial surgical resection was performed, the tissues were screened for presence of somatic *TIE2* or *PIK3CA* mutations, as described [21].

Exclusion criteria included severe, concurrent and/or uncontrolled diseases (cardiopathy, diabetes, infection, HIV, hypertension...), concomitant CYP3A4 inhibitor/inducer intake, gastro-intestinal disorders that might modify sirolimus absorption, pregnancy and lactation for women. Patients could not have had a surgical resection and/or sclerotherapy within 4 weeks prior to study entry. Exclusion criteria also included previous use of an mTOR inhibitor.

### Study protocol and treatment

This is a crossover trial using patient’s long-term clinical, biological and radiological history before sirolimus treatment as control [27]. Pre-treatment analysis included D-dimer and fibrinogen level measurements, and MRI studies using T1-weighted imaging without gadolinium chelate injection, T2-weighted imaging with Fat Saturation and/or STIR sequences in two orthogonal planes.

Sirolimus was started with a dose of 0.8 mg/m^2^ body surface, twice-a-day, using liquid solutions for children under the age of 12 years, and with a single dose of 2 mg/day, using a tablet, for older patients. All patients were seen every month for the first 3 months, then every 3 months in order to evaluate the signs and symptoms of the malformation, compliance and possible side-effects of the drug. After 1 week of treatment, sirolimus serum level was measured and daily sirolimus dose was adapted to reach a blood level between 10 to 15 ng/ml. If the patient experienced grade 3–4 toxicities, sirolimus dose was decreased. Hemogram, liver and renal function, coagulation parameters and thyroid tests were monitored every 3 months.

Treatment was suspended in cases of severe toxicity, patient and/or parents who refused to continue, and patients who did not experience any benefit after 3 months of therapy. For all patients enrolled in this study, a follow-up is planned for 5 years.

The primary endpoint was efficacy and safety of sirolimus at 12 months. Efficacy of the medication was evaluated based on physical (clinical size, colour and consistency of the lesion), functional (organ dysfunction, mobility restraint, pain, bleeding, oozing, repetitive infections), biological (measurement of coagulation parameters) and radiological response, as well as on quality of life (QoL) questionnaire. Clinical examination and measurement of lesions were performed at each consultation. Evaluation of pain was based on visual analog scale (VAS) for adult and on the faces pain scale for children, both ranging from 0 (no pain) to 10 (excessive pain). Radiological evaluation was based on the comparison between the pre-study MRI and the MRI performed at one-year follow-up. Imaging analysis was completed by using the ITK-SNAP_3–0_ software from the DICOM source images, to obtain an objective quantification of the volume of the malformation [28, 29]. This program required a manual or semi-automatic contouring of the lesions on the MRI slices, which subsequently generated a 3-dimensional image and volume calculation. QoL was based on a questionnaire modified from Medical Outcomes Study 36-Item Short Form (MOS SF-36) for adults. Specific questionnaires from “Pediatric Quality of Life Inventory” (PEDSQL) were used for infants from age 5–7 years, 8–12 years and 13–18 years, and a corresponding questionnaire for their respective parents (www.pedsql.org). Moreover, a global self-evaluation percentage (0% = no change to 100% = symptom free; improvement was considered as weak between 0 and 20%, moderate between 20 and 50% and strong when superior to 50%) was recorded for each patient and/or parents at each consultation. Coagulation changes (D-dimer and fibrinogen levels) were also recorded. Adverse events were assessed according to the Common Terminology Criteria for Adverse Events (CTCAE) version 3.0.

Response was defined as follows:

- Complete response (CR), defined as a complete disappearance of the lesion (clinical and/or radiological), of the symptoms and normalisation of QoL.
- Partial response (PR), defined as a reduction of ≥20% in size of the vascular lesion (clinical and/or radiological), improvement of symptoms and/or QoL.
- Absence of response (AR), defined as a progressive disease (increase in size, symptoms and decreased QoL) or disease stability (reduction of < 20% in size and no improvement of symptoms and QoL).

### Statistical analysis

Statistical analyses were performed using the SPSS version 24.0 [30]. Paired sample t-tests were applied with a significance threshold of 0.05.

## Results

Nineteen patients, 12 females and 7 males, aged from 3 to 64 years (median: 15 years) were enrolled between January 2011 and January 2015. Six patients had a LM, two had a generalized lymphatic anomaly (GLA), seven had a VM, one had a capillary venous malformation (CVM), two had KTS and one had PHTS. Characteristics of patients are described in Table 1.

**Table 1:** Characteristics of patients before and after 1 year of sirolimus treatment

	Sex Age	Type and mutation	Localisation	Previous treatment	Symptoms and laboratory findings	Duration of sirolimus	Improvement
#1	F 11y	Sporadic LM	Cervico-facial with laryngeal extension	Surgery Antibiotherapy	- Physical deformation - Speech trouble - Infections (3/6 months) - Poor quality of life	34 months, stopped with non-EBV lymphoma diagnosis	- Physical improvement - Functional improvement (speech) - Reduction of Infection - Quality of life moderately improved - Malformation reduction on MRI - Not evaluable with ITK-SNAP_3–0_
#2	F 16y	Sporadic LM Somatic PIK3CA mutation	Retro-orbitary and nose ala	Surgery Sclerotherapy Antibiotherapy	- Physical deformation - Eye opening difficulty - Continuous pain (VAS 5) with painful crisis (1–2 weekly) - Infections (1x/3 months) - Poor quality of life	13 months, stopped due to possibility for sclerotherapy	- Physical improvement - Functional improvement (eye opening) - Reduction of pain (VAS 4) and painful crisis - No infection within 13 months - Quality of life moderately improved - No malformation reduction on MRI - 0.6 cm^3^ (6.7%) volume reduction on ITK-SNAP_3–0_
#3	M 3y	Sporadic LM	Cervico-facial with parotid and tongue invasion	Surgery Antibiotherapy	- Physical deformation (tongue enlargement) - Speech and mastication trouble - Infections (1x/3 month) - Poor quality of life	28 months, ongoing	- Physical improvement - Functional improvement (speech and mastication) - No infection within 28 months - Quality of life strongly improved - Malformation reduction on MRI - 4 cm^3^ (2%) volume reduction on ITK-SNAP_3–0_
#4	F 8y	Sporadic LM	Right lower limb, buttock, pelvis, abdomen	Surgery Sclerotherapy Antibiotherapy	- Physical deformation (limb enlargement) - Walking, sitting and standing trouble - Continuous pain (VAS 5) with painful crisis at defecation - Daily rectal oozing - Infections (1x/2 months) - Poor quality of life	15 months, ongoing	- Functional improvement (walking and standing) - Reduction of pain (VAS 1) - Disappearance of rectal oozing - No infection within 15 months - Quality of life strongly improved - Radiological progression on MRI - Not evaluable with ITK-SNAP_3–0_
#5	M 5y	Sporadic LM	Diffuse, pulmonary, splenic, vertebral	Antibiotherapy	- Respiratory insufficiency (continuous oxygenotherapy) - Daily hemoptysis - Infections (1x/month) - Altered growth - Poor quality of life	41 months, ongoing	- Functional improvement (decreased oxygen-dependency) - Reduction of hemoptysis - Reduction of infection (2x/year) - Quality of life moderately improved - No size reduction on MRI - Not evaluable with ITK-SNAP_3–0_
#6	F 10y	Sporadic LM Somatic PIK3CA mutation	Right lower limb, buttock, pelvis, abdomen	Surgery Sclerotherapy Antibiotherapy	- Physical deformation (right foot enlargement) - Walking, sitting and standing trouble - Continuous pain (VAS 6) with painful crisis (5–10/month) - Infections (1x/2 months) - Poor quality of life	32 months, ongoing	- Physical improvement - Functional improvement (walking, sitting and standing) - Reduction of pain (VAS 1) - Reduction of infections (2x/year) - Quality of life strongly improved - Size reduction on MRI - Not evaluable with ITK-SNAP_3–0_
#7	M 4y	GLA PIK3CA mutation	Right lower limb, lower back, abdomen, buttock	LMWH Antibiotherapy	- Physical deformation (limb enlargement) - Walking and standing difficulties - Infection (1x/2 month) - Poor quality of life	48 months, ongoing	- Physical improvement - Functional improvement (walking and standing) - Reduction of infection (2x/year) - Quality of life strongly improved - Radiological progression on MRI - Not evaluable with ITK-SNAP_3–0_
#8	F 9y	GLA TIE2 mutation	Left lower limb, buttock, bones, uterine, pelvis, abdomen	LMWH Antibiotherapy	- Physical deformation (limb enlargement) - Sitting and walking difficulties - Continuous pain (VAS 7) with painful crisis (1–5/month) - Daily gynecological oozing - Infections (1x/month) - Poor quality of life	44 months, ongoing	- Physical improvement - Functional improvement (sitting and walking) - Disappearance of pain (VAS 0) - Disappearance of gynaecological oozing - Disappearance of infection - Quality of life strongly improved - Malformation reduction on MRI - 120 cm^3^ (7.6%) volume reduction on ITK-SNAP_3–0_
#9	F 64y	Sporadic VM Somatic PIK3CA mutation	Left lower limb, buttock, perineum, colon, spleen, liver, vagina	Surgery LMWH Elastic contention	- Physical deformation (limb enlargement) - Dressing, standing and walking trouble - Continuous pain (VAS 10) with painful crisis (10–20/month) - Daily gynaecological bleeding - Poor quality of life - Elevated D-dimer levels (5524 ng/ml) - Low fibrinogen levels (127 mg/dl)	35 months, ongoing	- Physical improvement - Functional improvement (dressing and walking) - Disappearance of gynaecological bleeding - Reduction of pain (VAS 5) - Quality of life strongly improved - No malformation reduction on MRI - 118 cm^3^ (17.2%) volume reduction on ITK-SNAP_3–0_ - Decreased D-dimer levels (1799 ng/ml) - Fibrinogen levels normalization (254 mg/dl)
#10	F 20y	Sporadic VM Somatic TIE2 mutation	Left upper limb	Surgery Sclerotherapy LMWH Elastic contention	- Left upper limb mobilisation difficulties - Continuous pain (VAS 7) and painful crisis (5–10/month) - Poor quality of life - Elevated D-dimer levels (1200 ng/ml) - Normal fibrinogen levels (274 mg/dl)	1 month, stopped for grade 3 mucositis	- No functional improvement - No pain reduction - No quality of life improvement
#11	M 18y	Sporadic VM Somatic TIE2 mutation	Right upper limb	Surgery Sclerotherapy LMWH Elastic contention	- Physical deformation (Right hand deformed) - Wrist and thumb mobility difficulties - Continuous pain (VAS 3) with daily painful crisis - Poor quality of life - Elevated D-dimer levels (2766 ng/ml) - Normal fibrinogen levels (235 mg/dl)	2 months, stopped for grade 3 mucositis	- Functional improvement (wrist and thumb mobilisation) - Reduction of pain (VAS 2) and painful crisis (1-2x/week) - Quality of life strongly improved
#12	M 54y	Sporadic VM Somatic TIE2 mutation	Right lower limb, buttock, lower back	Surgery Sclerotherapy LMWH Elastic contention	- Physical deformation (buttock asymmetry) - Sitting, standing and walking difficulties - Continuous pain (VAS 3) with painful crisis (5–10/month) - Poor quality of life - Elevated D-dimer levels (3174 ng/ml) - Normal fibrinogen levels (307 mg/dl)	48 months, ongoing	- Physical improvement - Functional improvement (walking, sitting position, no need for elastic contention) - Reduction of pain (VAS 1) - Quality of life strongly improved - No malformation reduction on MRI - 40 cm^3^ (2.4%) volume reduction on ITK-SNAP_3–0_ - - Decreased D-dimer levels (1908 ng/ml)
#13	F 19y	Multifocal sporadic VM Somatic TIE2 mutation	Thorax, back, 4 limb	Surgery Sclerotherapy LMWH Elastic contention	- Muscular weakness, difficulties in walking, writing, doing sport - Continuous pain (VAS 8) with painful crisis (1–5/month) - Poor quality of life, severe mood alteration - Elevated D-dimer levels (7587 ng/ml) - Normal fibrinogen levels (319 mg/dl)	28 months, ongoing	- Functional improvement (walking and writing) - Reduction of pain (VAS 5) - Quality of life moderately improved - Radiological progression on MRI - 0.1 cm^3^ (0.5%) volume reduction on ITK-SNAP_3–0_ - Decreased D-dimer levels (1048 ng/ml)
#14	M 39y	Sporadic VM Somatic TIE2 mutation	Left parieto-temporal region	Surgery LMWH	- Continuous pain (VAS 8) and frequent headache - Poor quality of life, severe mood alteration - Normal D-dimer levels (330 ng/ml) - Normal fibrinogen levels (382 mg/dl)	1 month, stopped spontaneously	Patient not evaluable
#15	F 23y	Sporadic VM Somatic PIK3CA mutation	Right ankle	Surgery Sclerotherapy LMWH Antalgic Elastic contention	- Physical deformation (Ankle enlargement) - Ankle mobility and walking difficulties - Continuous pain (VAS 7) with painful crisis (1–5/month) - Poor quality of life - Elevated D-dimer levels (1350 ng/ml) - Normal fibrinogen levels (315 mg/dl)	32 months, ongoing	- Physical improvement - Functional improvement (walking improvement, no need for elastic contention) - Reduction of pain (VAS 2) - Quality of life strongly improved - Malformation reduction on MRI - 1.7 cm^3^ (12.1%) volume reduction on ITK-SNAP_3–0_ - D-dimer levels normalization (460 ng/ml)
#16	F 9y	CVM No TIE2 mutation	Pelvis, uterine and rectal extension	LMWH Exacyl Transfusion	- Physical deformation - Sitting, standing and walking troubles - Daily rectorrhagia - Poor quality of life - Elevated D-dimer levels (1292 ng/ml) - Normal fibrinogen levels (356 mg/dl)	13 months, ongoing	- Functional improvement - Decreased rectal bleeding intensity - Decrease transfusions - Quality of life strongly improved - Malformation reduction on MRI - 37.2 cm^3^ (34.5%) volume reduction on ITK-SNAP_3–0_ - Decreased D-dimer levels (968 ng/ml)
#17	F 14y	KTS Somatic PIK3CA mutation	Right thigh, buttock, abdomen, vagina	Surgery Sclerotherapy Antalgic Antibiotherapy	- Physical deformation (thigh enlargement and abdomen asymmetry) - Muscle weakness, sitting and walking trouble - Gynaecological bleeding (2x/week) - Daily abdominal oozing - Infections (1x/3 month) - Poor quality of life - Elevated D-dimer levels (1685 ng/ml) - Normal fibrinogen levels (371 mg/dl)	26 months, stopped for surgery eligibility	- Physical improvement (thigh volume reduction and improved coloration) - Functional improvement (disappearance of mobility trouble) - Reduction of bleeding - Disappearance of oozing - Disappearance of infections - Quality of life moderately improved - Malformation reduction on MRI - 68 cm^3^ (18.8%) volume reduction on ITK-SNAP_3–0_ - Decreased D-dimer levels (800 ng/ml)
#18	F 30y	KTS No TIE2 or PIK3CA mutation	4 limbs, trunk	Surgery LMWH Antibiotherapy Elastic contention	- Physical deformation (left hemi-body hypertrophy) - Continuous pain (VAS 6) with painful crisis (> 20/month) - Daily oozing - Infections (1x/3 month) - Poor quality of life - Elevated D-dimer levels (5609 ng/ml) - Normal fibrinogen levels (414 mg/dl)	16 months, stopped due to pain reappearance	- Reduction of pain (VAS 2) - Reduction of oozing - Reduction of infections - Quality of life moderately improved - No malformation reduction on MRI - Not evaluable with ITK-SNAP_3–0_ - Decreased D-dimer levels (951 ng/ml)
#19	M 16y	PHTS Mutation in PTEN	Right upper limb, shoulder	Sclerotherapy	- Physical deformation - Mobility difficulty - Continuous Pain (VAS 4) with painful crisis (1 monthly) - Poor quality of life - Elevated D-dimer levels (889 ng/ml) - Normal fibrinogen levels (410 mg/dl)	13 months, ongoing	- Physical improvement - Functional improvement (mobility) - Disappearance of pain (VAS 0) - Quality of life strongly improved - No malformation reduction on MRI - Not evaluable with ITK-SNAP_3–0_ - D-dimer levels normalization (459 ng/ml)

*F* female, *M* male, *y* years, *LM* lymphatic Malformation, *GLA* Generalized Lymphatic Anomaly, *VM* Venous Malformation, *CVM* Capillary Venous Malformation, *KTS* Klippel-Trenaunay Syndrome, *PHTS* PTEN Hamartoma Tumour Syndrome, *LMWH* Low Molecular Weight Heparin, *VAS* Visual Analogue Scale, *MRI* Magnetic Resonance Imaging

All but one patient had previously been treated with several sequences of sclerotherapies and/or surgical resection. All had severe symptoms varying from esthetic deformation (*n* = 15), chronic pain (VAS 3–10) refractory to conventional medications (*n* = 13), organ or mobility dysfunction (*n* = 17), recurrent infections (*n* = 10), bleeding (*n* = 4), and/or oozing (*n* = 4). All patients had a poor QoL. Of the 11 patients with a venous component, 10 (90%) had elevated D-dimer levels and 1 VM (#9) had low fibrinogen levels before sirolimus initiation.

Sixteen patients received sirolimus for at least 12 months; two stopped earlier because of grade 3 mucositis and one spontaneously decided to stop the study. Thirteen patients are still on treatment, with a follow-up ranging from 15 to 48 months.

### Efficacy

Sixteen patients were evaluated for efficacy after 1 year of sirolimus treatment (Table 2). A partial response was observed in all patients (*n* = 16/16). There was no complete response.

**Table 2:** Efficacy after 1 year of sirolimus treatment

Efficacy at 12 months FU (*n* = 16)	LM (*n* = 6)	GLA (*n* = 2)	VM (*n* = 4)	CVM (*n* = 1)	KTS (*n* = 2)	PHTS (*n* = 1)	Total
*Physical examination (n = 14)*	*n* = 4/5 (80%)	*n* = 2/2 (100%)	*n* = 3/3 (100%)	*n* = 0	*n* = 1/2 (50%)	*n* = 1/1 (100%)	*n* = 11/14 (78%)
*Organ or mobility dysfunction (n = 15)*	*n* = 6/6 (100%)	*n* = 2/2 (100%)	*n* = 4/4 (100%)	*n* = 1/1 (100%)	*n* = 1/1 (100%)	*n* = 1/1 (100%)	*n* = 15/15 (100%)
*Pain (n = 10)*	*n* = 3/3 (100%)	*n* = 1/1 (100%)	*n* = 4/4 (100%)	*n* = 0	*n* = 1/1 (100%)	*n* = 1/1 (100%)	*n* = 10/10 (100%)
*Bleeding (n = 4)*	*n* = 1/1 (100%)	*n* = 0	*n* = 1/1 (100%)	*n* = 1/1 (100%)	*n* = 1/1 (100%)	*n* = 0	*n* = 4/4 (100%)
*Oozing (n = 4)*	*n* = 1/1 (100%)	*n* = 1/1 (100%)	*n* = 0	*n* = 0	*n* = 2/2 (100%)	*n* = 0	*n* = 4/4 (100%)
*Repetitive infections (n = 10)*	*n* = 6/6 (100%)	*n* = 2/2 (100%)	*n* = 0	*n* = 0	*n* = 2/2 (100%)	*n* = 0	*n* = 10/10 (100%)
*Quality of life (n = 16)*							
- *No amelioration*	*n* = 0	*n* = 0	*n* = 0	*n* = 0	*n* = 0	*n* = 0	*n* = 0 (0%)
- *Moderate amelioration*	*n* = 3/6 (50%)	*n* = 0	*N* = 1/4 (25%)	*n* = 0	*n* = 2/2 (100%)	*n* = 0	*n* = 6/16 (37.5%)
- *Strong amelioration*	*n* = 3/6 (50%)	*n* = 2/2 (100%)	*n* = 3/4 (75%)	*n* = 1/1 (100%)	*n* = 0	*n* = 1/1 (100%)	*n* = 10/16 (62.5%)
*Reduced coagulopathy (n = 8)*	n/a	n/a	*n* = 4 /4 (100%)	*n* = 1/1 (100%)	*n* = 2 /2 (100%)	*N* = 1/1 (100%)	*n* = 8/8 (100%)
*MRI (T1,T2 Fat Sat,STIR) (n = 16)*							
- *Progression*	*n* = 1/6 (17%)	*n* = 1/2 (50%)	*n* = 1/4 (25%)	*n* = 0	*n* = 0	*n* = 0	*n* = 3/16 (19%)
- *Status quo*	*n* = 2/6 (33%)	*n* = 0	*n* = 2/4 (50%)	*n* = 0	*n* = 1/2 (50%)	*n* = 1/1 (100%)	*n* = 6/16 (37%)
- *Amelioration*	*n* = 3/6 (50%)	*n* = 1/2 (50%)	*n* = 1/4 (25%)	*n* = 1/1 (100%)	*n* = 1/2 (50%)	*n* = 0	*n* = 7/16 (44%)
*ITK-SNAP*_*3–0*_ *(n = 9)*							
- *Volume augmentation*	*n* = 0	*n* = 0	*n* = 0	*n* = 0	*n* = 0	*n* = 0	*n* = 0/9 (0%)
- *No volume modification*	*n* = 0	*n* = 0	*n* = 1/4 (25%)	*n* = 0	*n* = 0	*n* = 0	*n* = 1/9 (11%)
- *Volume reduction*	*n* = 2/2 (100%)	*n* = 1/1 (100%)	*n* = 3/4 (75%)	*n* = 1/1 (100%)	*n* = 1/1 (100%)	*n* = 0	*n* = 8/9 (89%)

*FU* Follow-Up, *LM* Lymphatic Malformation, *GLA* Generalized Lymphatic Anomaly, *VM* Venous Malformation, *CVM* Capillary Venous Malformation, *KTS* Klippel-Trenaunay Syndrome, *PHTS* PTEN Hamartoma Tumour Syndrome, *n/a* not applicable

#### Lesional change

**Fig. 1:**
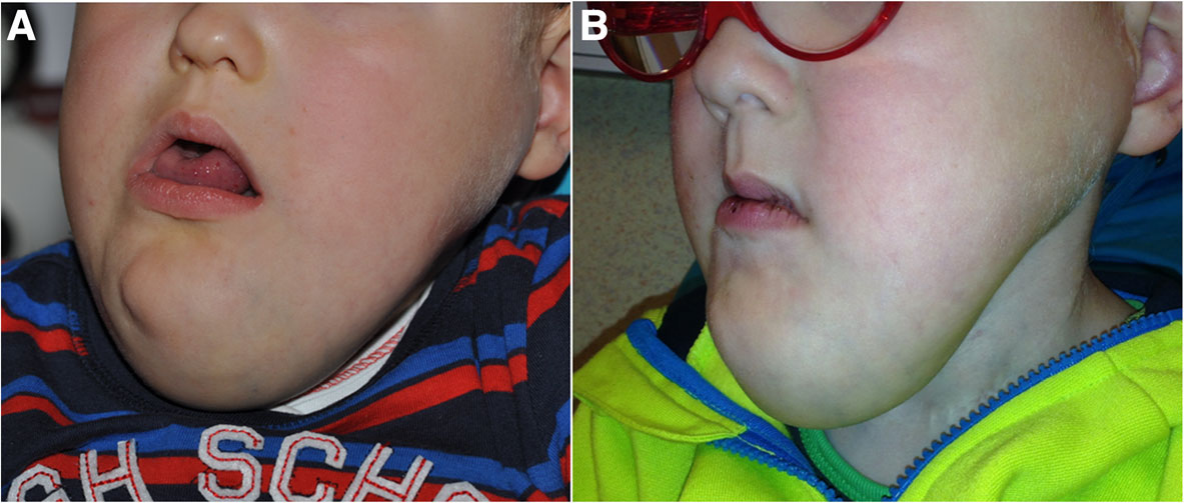
Patient #3 with a cervico-facial microcystic LM before initiation of sirolimus (**a**) and after 12 months of treatment (**b**)

**Fig. 2:**
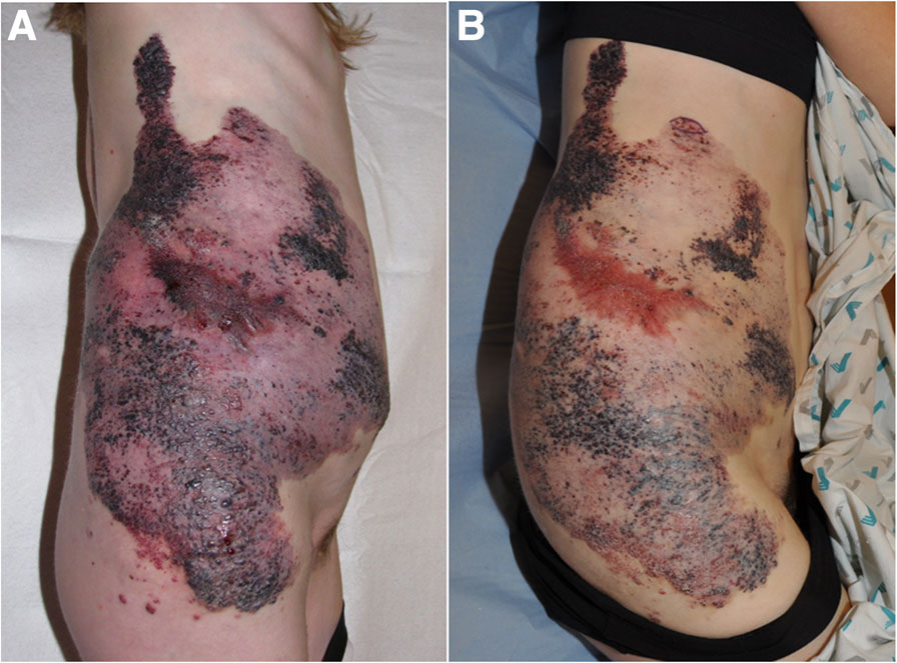
Patient #17 with a capillaro-lymphatico-venous malformation with hypertrophy (Klippel-Trenaunay syndrome) before initiation of sirolimus (**a**) and after 6 months of treatment (**b**)

In patients with visible vascular malformations sirolimus resulted in a noticeable improvement in appearance compared to previous photographs in 11 patients at 1 year time-point (Figs. 1 and 2) resulting in an clinical response rate of 78%. This improvement appeared within 3 months from the start of sirolimus in all of these patients and persisted at 6- and 12-month evaluation points. No clinical disappearance of the lesion was observed but the volume reduction induced by sirolimus allowed sclerotherapy for patient #2 and surgical resection for patient #17. The patient who stopped sirolimus after 2 months because of grade 3 mucositis (#11) did not have any physical improvement during this short period.

#### Functional change

All patients experienced improvement in mobility or organ function, reduction of pain, bleeding or oozing, or reduction or cessation of infections, resulting in a 100% general improvement rate. These appeared within 3 months from the start of sirolimus and were maintained. Similarly, patient #11 (with a limited 2-month-treatment) experienced improvement in mobility.

**Fig. 3:**
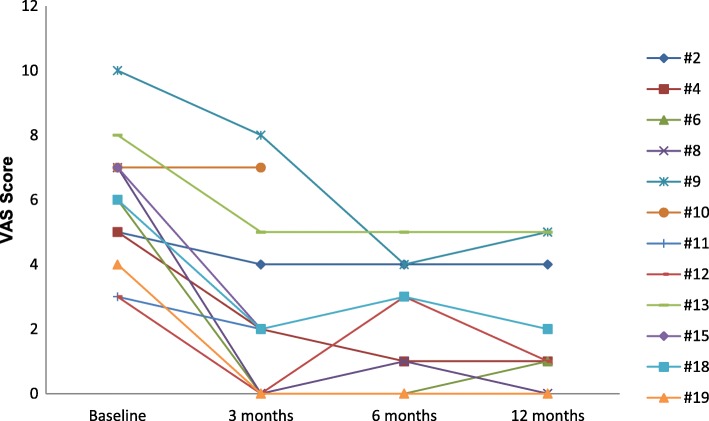
Visual Analogue Scale (continuous pain) results for all patients

*Pain* improved in all evaluable patients (*n* = 10) (Fig. 3). These patients suffered from chronic pain (VAS ranging from 3 to 10) with frequent painful crises commonly reaching at least VAS 8. Median VAS score for baseline continuous pain decreased from 6 to 2 at 3, 6 and 12 months follow-up and this improvement persisted at 6 and 12 months follow-up. The median frequency of painful crises decreased from 5/month to 1/month (*p* = 0.001). Interestingly, pain recurred within 72 h in two patients, 1 with LM (#6) and 1 with VM (#9), after sirolimus cessation for grade 2 headache and fatigue, respectively, and reduced again rapidly after sirolimus reintroduction. For one patient with extensive lower limb VM (#12), pain disappeared completely after 2 weeks of treatment but reappeared after 6 months despite correct sirolimus blood level and an increased dosage (3 mg/day). After a 2-month therapy-gap, sirolimus reintroduction reduced pain again. Patient #11 (with a limited 2-month-treatment) also experienced pain reduction.

*Bleeding* was a common sign in four patients. It reduced significantly in three patients (#5, #16, #17) and stopped completely in one patient (#9). After a few months, patients #9 and #16 stopped the treatment during 2 weeks due to grade 2 fatigue, and bleeding resurged rapidly (in less than 72 h). Sirolimus reintroduction stopped the bleeding in less than 48 h in both patients.

*Infections and oozing* also improved in all patients who frequently experienced them prior to treatment. Median frequency of infections per 6 months decreased from 3 to 1. The four patients with oozing (#4, #8, #17, #18) experienced a rapid reduction in intensity and frequency with sirolimus.

#### Quality of life

After 1 year of sirolimus treatment, QoL improved in all patients (*n* = 16/16). Already after 3 months of treatment, moderate (20–50%) or strong (> 50%) QoL improvement was seen in 6 and 10 patients, respectively. This improvement continued at 6 and 12 months time-points for all patients. Patient #12 (see above) who had a strong initial improvement at 3 months, showed a decrease at 6 months due to pain resurgence, despite continuation and a dose-increase of sirolimus. After 2-month treatment arrest and sirolimus reintroduction, QoL improved again. Interestingly, patient #11 who stopped sirolimus after 2 months because of grade 3 mucositis, presented a strong QoL improvement during this period.

#### Coagulopathy

**Fig. 4:**
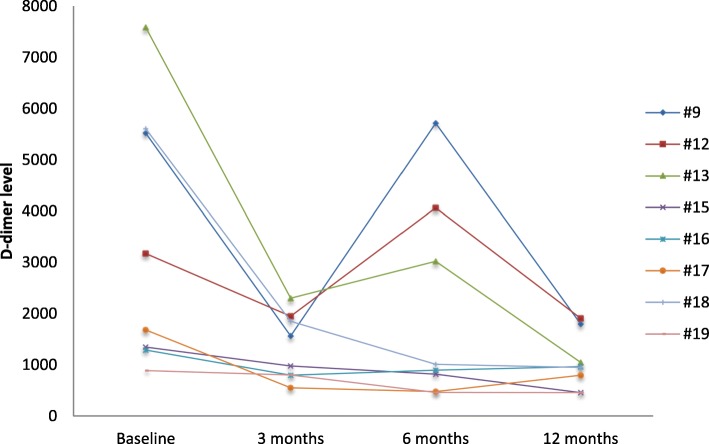
D-dimer levels

Sirolimus decreased coagulation abnormalities. Out of the 11 patients with a venous component, 8 reached 12 months of treatment (all had pre-treatment D-dimer elevations). D-dimer levels decreased in all patients at 3 months and this reduction persisted at 12 months (*p* = 0.012) (Fig. 4). In two patients (#9, #12), D-dimer levels showed a temporary fluctuation that was correlated with resurgence of symptoms.

#### Radiological aspect

**Fig. 5:**
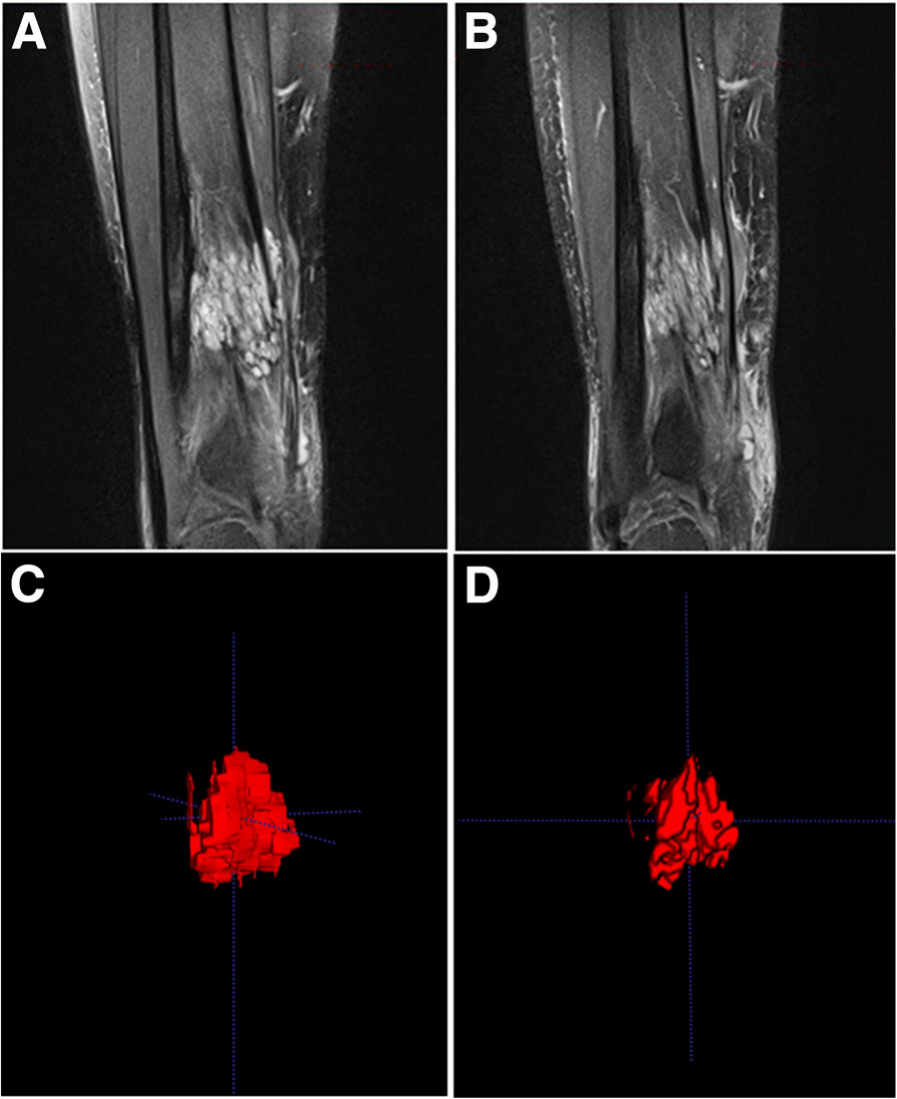
Classical MRI sequences and 3D-volumes of the malformation (patient #15) before initiation of sirolimus (**a**, **c**) and after 12 months of treatment (**b, d**). Notice volume reduction from 14 cm^3^ to 12,3 cm^3^

Among the 16 patients radiologically evaluated at 12 months of treatment, T2-MRI-weighted sequences showed a size reduction in 7 of them (43.7%): three LM, one GLA, one VM, one CVM and one KTS. Despite clinical improvement, radiological progression of their malformation was observed in three patients (18.7%): one LM, one GLA and one VM. On ITK-SNAP_3–0_ evaluation, eight patients (88.9%) showed reduction of their malformation (two LM, one GLA, three VM, one CVM, and one KTS) (Fig. 5). Only patient #13 did not present significant volume reduction (reduction only 0.1 cm^3^ (0.5%)). None of the nine patients evaluated with the ITK-SNAP segmentation program presented an augmentation of the malformation volume. Seven patients (43.7%) had to be excluded from this quantitative evaluation because of very infiltrative and/or multifocal disease compromising an adequate and reliable contouring of the lesions.

### Safety

Sirolimus was well tolerated (Table 3): headache, skin rash, mucositis, fatigue and diarrhea were the most frequent grade 1–2 adverse events. All were easily manageable with symptomatic treatment or temporary arrest. Three patients stopped sirolimus for a few days due to grade 2 headache (#6) or grade 2 fatigue (#9, #16).

**Table 3:** Adverse events (CTCAE version 3.0)

Adverse events	Grade 1–2 n (%)	Grade 3–4 n (%)
Conjunctiva	1 (5.3%)	0
Pneumonitis/pulmonary infiltrates	1 (5.3%)	0
Neuropathy	1 (5.3%)	0
Skin cancer (*basal cell carcinoma*)	0	1 (5.3%)
Lymphoma	0	1 (5.3%)
Anemia	0	0
Thrombocytopenia	1 (5.3%)	0
Leucopenia	0	0
Hypercholesterolemia/hyperglycemia	0	0
Headache	11 (57.9%)	0
Arterial hypertension	1 (5.3%)	0
Diarrhea	7 (36.9%)	0
Nausea/vomiting	5 (26.4%)	0
Mucositis/stomatitis	7 (36.9%)	2 (10.6%)
Abnormal liver function tests	0	0
Rash	7 (36.9%)	0
Arthralgia	1 (5.3%)	0
Flu-like syndrome	6 (31.6%)	0
Fatigue	9 (47.4%)	0
Wound healing	2 (10.6%)	0
Weight loss	3 (15.8%)	0
Insomnia	4 (21.1%)	0

Mucositis was the most common grade 3 adverse event and led to definitive discontinuation of sirolimus in 2 patients (#10, #11) despite dose adjustment. One 64-year-old patient, with previous history of basal celle carcinomas, had a recurrence of basal cell carcinoma after 1 year of treatment (#9) and was cured by surgical resection without sirolimus interruption. One patient was diagnosed with diffuse large B-cell lymphoma 34 months after treatment initiation (#1). This cancer was considered not to be related to sirolimus medication as it was not an EBV lymphoma.

## Discussion

This prospective phase II study confirms the overall efficacy and safety of sirolimus in patients with refractory-to-standard-care slow-flow vascular malformations. Nineteen patients were included in this trial, representing an important number of these severe cases. All our enrolled patients were highly symptomatic, had a poor QoL and had failed to respond to previous therapies, including surgery, sclerotherapy and various medications. Thus, their lesional areas contained scarred tissues, not only nascent malformation. Yet, sirolimus was clearly efficacious.

Sirolimus was highly effective in VM, LM and combined lesions, and resulted in a partial response in all patients, reducing symptoms and increasing QoL. This was indirectly confirmed by the fact that the majority of patients decided to continue sirolimus treatment even after study end-point due to the significant improvement. Our study also confirmed the reported data that sirolimus does not cure patients with vascular anomalies [22, 25, 26, 31].

The efficacy of sirolimus on pain, bleeding and oozing appeared within the first 3 months. Interestingly, the temporary arrest of sirolimus in three patients was associated with a rapid resurgence of pain and/or bleeding, and its reintroduction induced a reversibility. This impressive rapidity of action urges to consider sirolimus in life-threatening vascular malformations. Furthermore, sirolimus allowed interventional procedures that were initially considered unfeasible to be performed in two patients, suggesting that sirolimus could play a role as a pre-treatment before radical and curative interventional procedures of large complicated lesions. As most patients in this study had responded by 6 months, such a pre-treament period could be envisioned. Future clinical trials should be performed to evaluate efficacy of sirolimus on nascent malformations free of scaring.

Sirolimus was well tolerated, both in children and in adults. Adverse events were mostly grade 1 and 2, and easily manageable with symptomatic treatment or temporary arrest. Mucositis was the most common grade 3 adverse event. A young patient presented lymphoma after 34 months of treatment. Despite the fact that it is not possible to completely exclude an indirect role of sirolimus due to its immonusuppressive effect, we considered, after a multidisciplinary discussion, that the lymphoma was not directly caused by the medication since the lymphoma was not of EBV-type. Similarly, a 64-year old patient with a basal cell carcinoma after 1 year of treatment, had a previous history of basal cell carcinomas. Thus, it was not considered to be associated with the sirolimus treatment. Despite absence of direct correlation between sirolimus and these adverse events, follow-up of patients treated with mTOR inhibitor is mandatory and should include an annual dermatological control, and a haematological control (hemogram, liver, thyroid and renal function) every 3–6 months.

D-dimer level could appear as a potential predictive biomarker for efficacy, since in our study D-dimer levels were associated with sirolimus efficacy and symptomatology. Levels decreased in all of the evaluated patients with a venous component. Furthermore, in two patients (#9, #12) with initial sirolimus efficacy, symptoms reappeared and were associated with a re-increase in D-dimer levels. Further studies should evaluate early evolution of D-dimers in order to identify patients who mostly benefit from mTOR inhibition.

Despite largely favourable clinical responses, qualitative interpretation of MRI studies routinely used to evaluate clinical progression of vascular malformations, detected regression of lesions only in a minority of patients. The more sensitive ITK-SNAP_3–0_ evaluation unravelled a slightly higher response rate of a 2-to-34% reduction of malformation volume in 8 out of 9 patients at 12 months. This underscores the use of clinical examination, QoL questionnaire and biomarkers as the best tools to evaluate clinical response.

Our study is limited by the number of patients enrolled - extensive, refractory-to-standard-care VM, LM and complex vascular lesions are rare - and the heterogeneity of the malformations. Yet, they have a common pathophysiological basis: activation of the PIK3/AKT/mTOR signalling pathway, the target of sirolimus treatment. The results of this phase II study underscore the efficacy of sirolimus in these varied lesions.

Follow-up reached for some patients 48 months. Although too short to confirm long-term efficacy and safety of sirolimus, it gives a basis for such evaluation. As patients experienced clear amelioration of their quality of life, long-term treatment may become a rule. The best tools to measure efficacy thus also seem to be QoL questionnaire and pain with VAS (both with a subjective component). Both may though have confounding effects as many patients have been symptomatic since they were born. Initiation of sirolimus treatment often makes them realize the level of chronic pain they were used to. In patients with a venous component, biomarkers such as D-dimer levels are very helpful to monitor efficacy.

All these results are in concordance with our original report on VM and the few reports on LM and/or VM [22, 25, 26, 31]. Notably, Adams and coworkers performed a prospective two-center study with similar response criteria and results [31]. The repeated demonstration of efficacy urges larger phase III trials to be conducted, with enlargement of enrolment criteria.

## Conclusion

Our study confirms that sirolimus is an effective therapeutic option for symptomatic patients with extensive LM, VM and/or complex malformation for which surgery and sclerotherapy do not provide a satisfactory solution. The quality of life is enhanced and pain is reduced. Indications may need to be enlarged, and studies performed on nascent lesions.
